# Contraction ratio of multifidus and erector spinae muscles in unilateral sacroiliac joint pain: A cross-sectional trial

**DOI:** 10.1038/s41598-024-84283-6

**Published:** 2025-01-11

**Authors:** Omar M. Mabrouk, Khaled E. Ayad, Doaa A. Abdel Hady

**Affiliations:** 1 MSK Sonographer, Department of Basic Science, Faculty of Physical Therapy, Deraya University, Minia, Egypt; 2 Department of Orthopaedic Physical Therapy, Deraya University, Minia, Egypt; 3 Department of Physical Therapy for Women’s Health, Faculty of Physical Therapy, Deraya University, Minia, Egypt

**Keywords:** Contraction ratio, Back muscles, Erector spinae, Multifidus, Unilateral sacroiliac joint pain, Diseases, Health care

## Abstract

Sacroiliac joint (SIJ) pain is one of the most prevalent reasons for disability, it affects the contraction ratio of the muscles of the back. Imaging is critical for diagnosing back muscles. The purpose of this study was to look at changes in the muscle contraction ratio of the lumbar multifidus (LM) and erector spinae (ES) in unilateral SIJ pain. This research included 60 individuals (30 with unilateral SIJ pain and 30 healthy people (who served as matching controls). The contraction ratio of back muscles such as ES and LM was assessed using real-time ultrasonography, and the results were compared between the affected and non-affected sides in patients with unilateral SIJ pain, and healthy participants as well. In the study group, the contraction ratio of ES and LM muscles on the non-affected side was significantly higher than on the affected side (p < 0.05). as well as a significant increase in contraction ratio of the ipsilateral side (affected matched control side) LM of the healthy group compared with that of the non-affected side of the study group (p < 0.001), while there was no significant difference in contraction ratio of the contralateral (unaffected matched control side) ES of the healthy group compared with that of the non-affected side of the study group. The results of this trial demonstrate that patients with unilateral SIJ pain exhibited a substantially lower muscle contraction ratio in the ES and LM of the affected side than the non-affected side in the study group, as well as a significant increase in the contraction ratio of the ES and MF on the ipsilateral side of the control group compared with that of the study group. However, there was no significant change in the contraction ratio of the contralateral ES in healthy individuals compared to the non-affected side of the study group. The findings of the study may help in designing an appropriate exercise program to deal with patients with SIJ pain.

## Introduction

The sacrum and two innominate pelvic bones create the sacroiliac joints (SIJ)^1^. The joint is commonly described as a strong, auricular-shaped, diarthrodial synovial joint^2^. The SIJ contains multiple muscles that act together to compress and regulate it, hence increasing pelvic stability and allowing for optimal load transmission across the pelvis across a variety of functional tasks^3^.

The primary function of SIJ is shock absorption and torque conversion, together with pelvic stabilization^4^. SIJ pain is reputed to be a source of lower back pain (LBP), with a worldwide prevalence reported to range from 0.4 to 35%, but the SIJ is the most overlooked source of LBP^5^. While no muscles are designed to act on the SIJ to produce active movements, the joint is still surrounded by some of the largest and most powerful muscles in the body^6^. These muscles include the erectrospinae(ES), quadratus lumborum, piriformis, gluteal, psoas, abdominal obliques, and hamstrings, but they do not act directly on the SIJ^7^.

ES and LM provide sacral flexion and force closure of the SIJ with deep abdominals^8^. CT, MRI, and rehabilitative ultrasound imaging (RUSI) are the three most popular diagnostic procedures used to evaluate paraspinal muscles. Muscle thickness, density, and contraction are all important factors to consider while assessing muscles via RUSI^9^. SIJ pain is also known as a change in the position of the SIJ surfaces caused by recurrent stress and sustained by compressed and elastic forces from both muscles and ligaments^10^. SIJ pain is characterized by altered biomechanical characteristics, neuronal compression, and muscular spasms^11^.

Ultrasonography measurements of muscle thickness variation precisely represent muscular activity at low levels (< 40% maximal voluntary contraction). Thus, muscular contraction ratio (CR), as a possible indication of muscular-tissue state (defined as contracted thickness or resting thickness of muscles), has recently been proposed for estimating muscle activity^12^.

Thus, it was hypothesized that people with SIJ pain would have a lower muscle contraction ratio of the lumbar multifidus (LM) and erector spinae (ES) in unilateral SIJ discomfort. To our knowledge, investigations have demonstrated the thickness of the external oblique, rectus abdominis, LM, internal oblique, and transversus abdominis in patients with SIJ discomfort and there is a reduction in resting muscle thickness, which demonstrates an altered motor pattern of the local muscle system and global muscular system^13^. SIJ problems can also be caused by changes in the resting muscle thickness of the latissimus dorsi and gluteal maximus ^14^. In addition, trunk movement patterns in women with LBP were investigated^15^. There has been no investigation into the contraction ratio of muscles in SIJ dysfunction. So, the primary goal of this study was to analyze and evaluate changes in LM and ES thickness during contraction against rest (contraction thickness ratio) among individuals with SIJ pain and healthy individuals.

## Materials & methods

### Study design

Cross-sectional research based on the STROBE declaration was carried out^16^. The Helsinki Declaration and human experimentation guidelines were evaluated and received ethical permission from the institutional review board at Deraya University’s Ethics Committee (approval number DCSR-01024–05). Prior to being included in the study, all participants were informed and given the opportunity to provide written consent. It was done from January 2024 to, April 30, 2024.

### Participants and recruitment

**Fig. 1:**
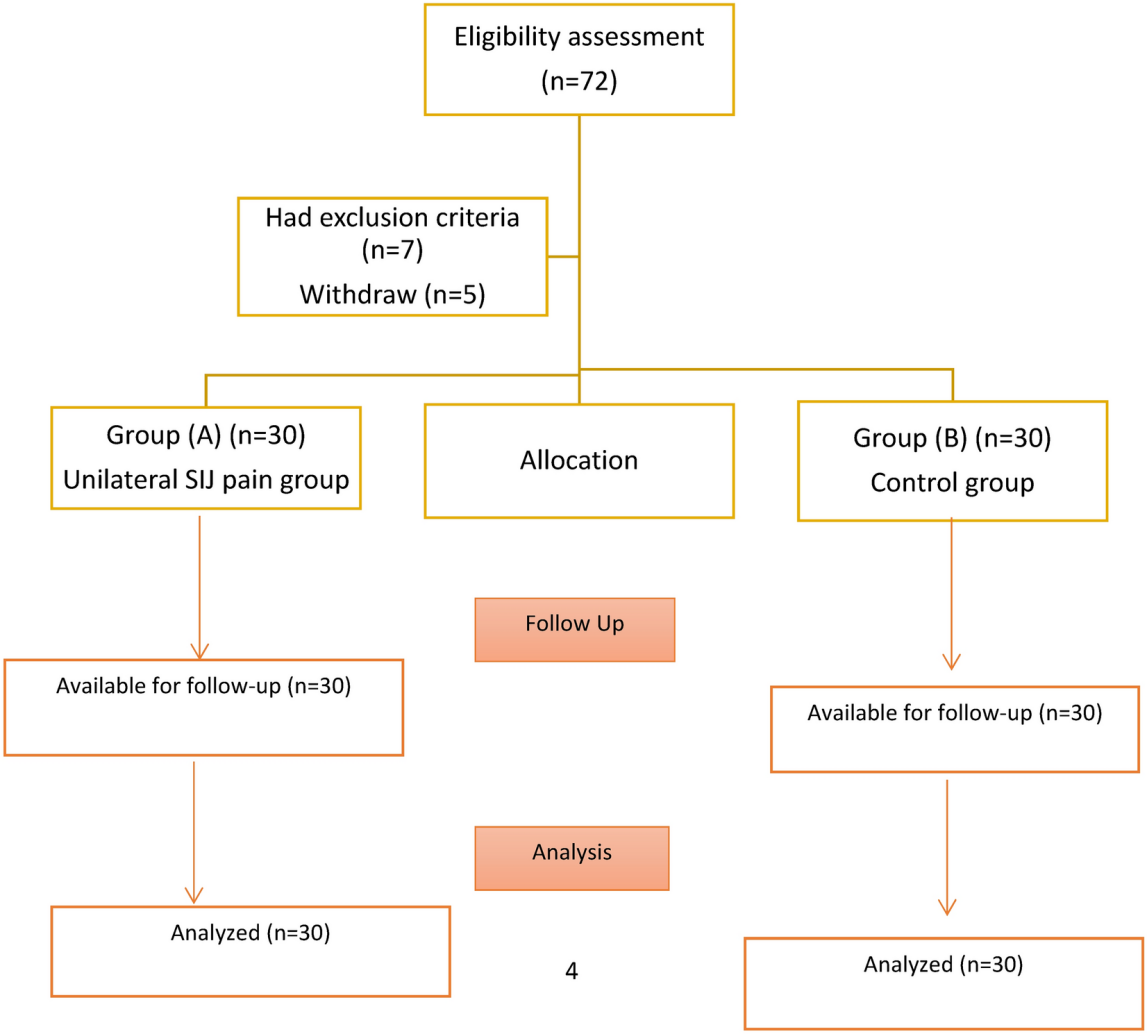
Flow chart of the study.

A total of 60 participants were recruited from the orthopedic outpatient clinic depending on an orthopedist’s diagnosis and recommendation. The study comprised thirty participants from each group. Participants with unilateral SIJ pain were assigned to "group A," whereas normal participants were assigned to control group “group B." Fig. 1

### Sample size

Sample size calculation was performed using G*POWER statistical software (version 3.1.9.2; Franz Faul, Universitat Kiel, Germany), expecting a large difference between groups and revealing that the required sample size was 30 subjects in each group. The calculation is made with α = 0.05, power = 85%, and effect size = 0.8. at Kiel, Germany), expecting a large difference between groups and revealing that the required sample size was 30 subjects in each group. The calculation is made with α = 0.05, power = 85%, and effect size = 0.8.

## Evaluation methods

### Inclusion and exclusion criteria

Participants were diagnosed with non-specific unilateral SIJ pain according to orthopedist diagnoses and recommendations, and they responded positively to at least three of four diagnostic tests (Gaenslen’s test, compression test, thigh thrust test, and Patrick’s test), pain form six months in the lower back and buttock in one side, with healthy participants serving as the control group in regards to age, weight, height, and body mass index (BMI).

According to the subsequent criteria: Their ages varied from 25 to 35 years, and their BMI was 20–25 kg/m2. In Group A, 30 patients complained of unilateral SIJ pain. This research includes 30 healthy participants in Group B. None of the healthy individuals had any prior history of musculoskeletal disorders, SIJ pain or a history of pain in the last year, or had any history of spinal operations, arthritis, neurological disorders, or lower limb problems (such as fractures, ankle or knee sprains, chronic ankle instability, lumbar pain or a groin, pubic or hip illnesses such as a hernia of the inguinal canal, the pubic region bone problem, or femoroacetabular impignement, respectively). The occurrence of radicular symptoms (like electrical pain or a burning sensation) as measured by the active straight leg test, the presence of lumbopelvic congenital diseases, and individuals’ incapacity to follow research instructions were all excluded from the trial.

### Ultrasound imaging assessment

All ultrasound examinations were performed by a sonographer, and those performing the ultrasound assessments were blinded to participants in groups. An ultrasound device (Mindray DP10, curvilinear probe, with a frequency of 2–5 MHz, serial number: bn-75013216, China) was used to measure changes in LM and ES muscle thickness during contraction relative to rest (contraction thickness ratio).

Before taking measurements, each subject was informed and practiced the activity to be done until they could accurately execute it.

#### Lumbar multifidus contraction ratio

**Fig. 2:**
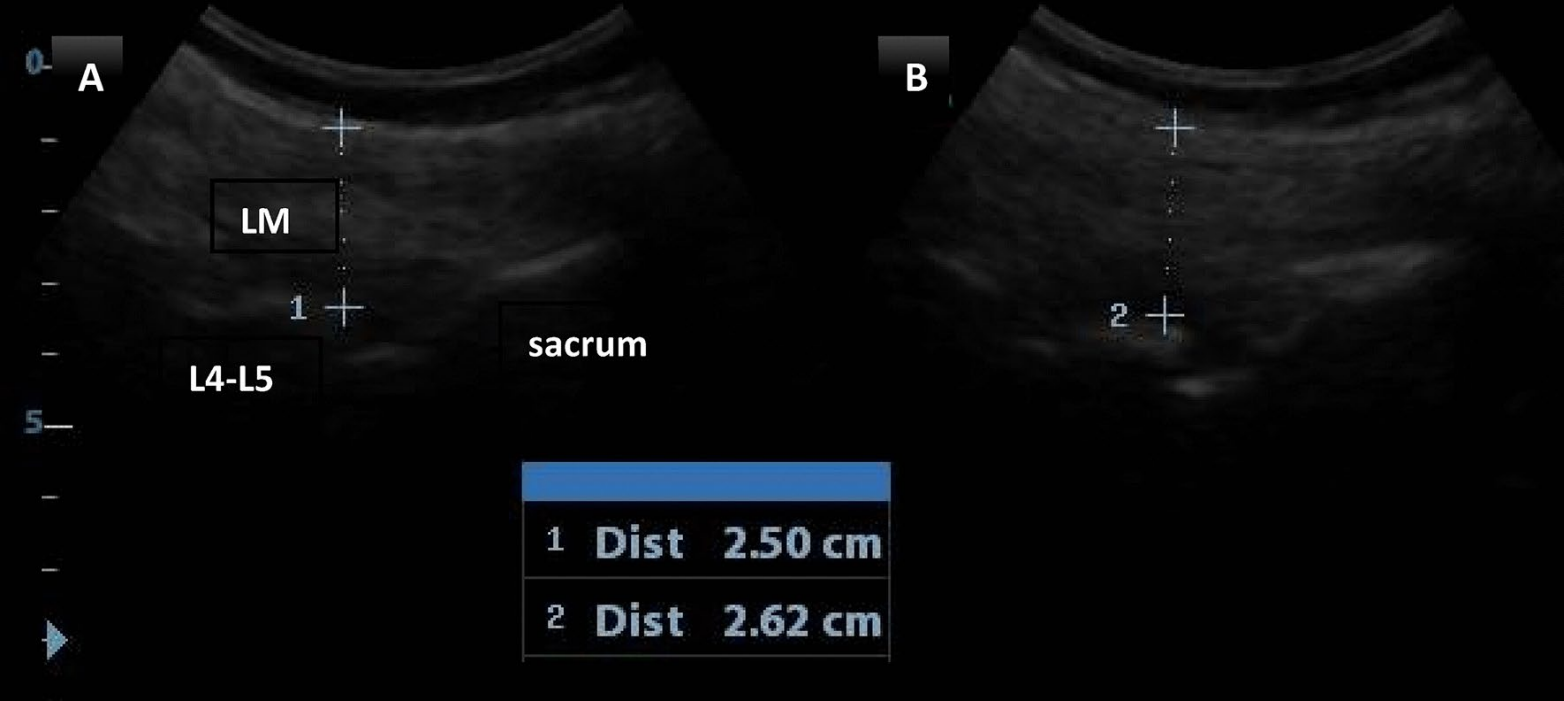
US measurement of LM contraction thickness (**A**) affected side (**B**) non affected side.

To measure the deep LM contraction ratio, each individuals in the prone position with an under-abdomen pillow to flatten the lumbar curvature and their head centered in the midline. The transducer was placed at the level of the L4 spinous process, subsequently moved little laterally and slightly rotated medially until the L4/5 apophyseal joint was visible on the screen. The thickness at rest and contraction when the patient was directed to raise the opposite lower limb were determined^17^. Spots were put on the skin with a marker to ensure uniformity throughout the measurements. The job contractions lasted around 10 s, and in order to acquire US pictures, they were frozen on the screen and saved to the scanner for measurement of LM thickness throughout contraction with screen calipers^18^.Thickness was determined using a curvilinear probe in a sagittal section as the perpendicular distance from the highest point of the facet joint to the plane between the thoracolumbar fascia and the subcutaneous fat on each side^19^. Fig. 2

#### Erector spinae muscle contraction ratio

**Fig. 3:**
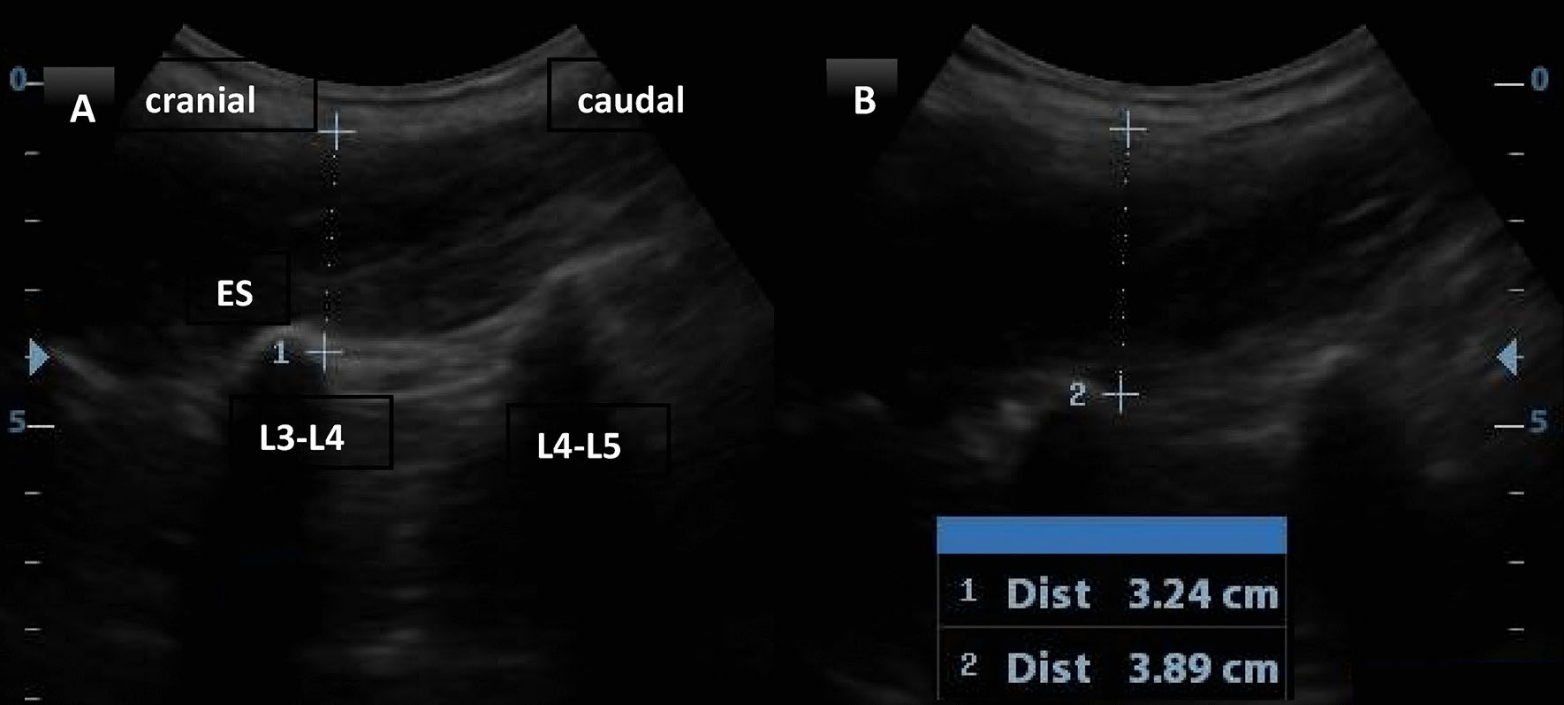
US measurement of ES contraction thickness (**A**) affected side (**B**) non affected side.

To determine the ES muscle contraction ratio, thickness at rest, and maximum isometric lumbar extension for 5 s, they were measured on each side at the level of the L3 vertebrae while lying prone^20^. The curvilinear probe was positioned longitudinally lateral to the spinous processes until transverse processes showed. Measurements were obtained on both sides, from the facet joint to the superficial fascial line^21^. The LM and ES thickness variations during contraction were measured in comparison to their rest thickness. The contraction thickness ratio (CTR) was computed as the percentage change between rest and exercise using the following equation: thickness contraction—thickness rest / thickness rest × 100. To minimize variability by around 50%^22^. This approach for measuring the LM using ultrasonography is reliable and valid^23^, and when ES was compared to the LM^17^, the mean of the three measurements was taken Fig. 3

US measurements show strong to excellent interrater (intraclass correlation coefficient (ICC) > 0.75) and intra-rater reliability (ICC > 0.90) among expert and rookie raters during the LM’s rest and contraction^24^. Additionally, (0.98–0.98) for ES muscle thickness and 0.994 for torque^20^.

### Statistical analysis

All analyses were performed by a statistician who was blinded to group assignment. An unpaired t test was conducted for comparison of subject characteristics between groups. A chi-squared test was used for comparison of sex and affected side distribution. The normal distribution of data was checked using the Shapiro–Wilk test. Levene’s test for homogeneity of variances was conducted to test the homogeneity between groups. An unpaired t test was conducted for comparison of the contraction ratio of erector spinae and multifidus between groups, and a paired t test was conducted for comparison between the affected and non-affected sides of the study group. The level of significance for all statistical tests was set at p < 0.05. All statistical measures were performed through the statistical package for social sciences (SPSS) version 25 for Windows.

## Results

### Subject characteristics

Subjects’ characteristics were demonstrated in Table 1. There was no significant difference between groups in age, BMI and sex distribution (p > 0.05).

**Table 1:** Basic characteristics of participants.

	Group A	Group B	MD	t- value	p-value
	Mean ± SD	Mean ± SD			
Age (years)	32.53 ± 5.09	31.47 ± 4.79	1.06	0.84	0.41
BMI (kg/m^2^)	24.52 ± 1.39	24.31 ± 1.27	0.21	0.61	0.54
Side of SJD					
a) Right side b) Left side	18 12				
Gender					
Male Female	10 (33%) 20 (67%)	10 (33%) 20 (67%)		(χ2 = 0)	1

SD, standard deviation; MD, mean difference; p-value, probability value.

### Comparison of contraction ratio of erector spinae and multifidus muscles between study and control groups

There was a significant increase in contraction ratio of ES and MF on the ipsilateral side of the control group compared with that of the study group (p < 0.001), as well as a significant increase in contraction ratio of contralateral MF muscle in the control group compared with that of the non-affected side of the study group (p < 0.001), while there was no significant difference in contraction ratio of contralateral ES in the control group compared with that of the non-affected side of the study group(Tables 2 and 3).

**Table 2:** Comparison of contraction ratio of the affected and ipsilateral sides of the erector spinae and multifidus between study and control groups:

Contraction /relaxation ratio	Study group Affected	Control group Ipsilateral		95% CI				
	Mean ± SD	Mean ± SD	MD	Lower limit	Upper limit	t- value	p value	Effect size
Erector spinae	0.11 ± 0.08	0.45 ± 0.09	-0.34	-0.39	-0.30	-15.42	0.001	3.99
Multifidus	0.12 ± 0.07	0.54 ± 0.11	-0.42	-0.47	-0.38	-17.95	0.001	4.56

SD, standard deviation; MD, mean difference; CI, confidence interval; p-value, probability value.

**Table 3:** Comparison of contraction ratio of the non-affected and contralateral -sides of the erector spinae, and multifidus between study and control groups:

Contraction /relaxation ratio	Study group Non affected	Control group Contralateral side		95% CI				
	Mean ± SD	Mean ± SD	MD	Lower limit	Upper limit	t- value	p value	Effect size
Erector spinae	0.39 ± 0.09	0.43 ± 0.10	-0.04	-0.09	0.01	-1.56	0.12	0.42
Multifidus	0.16 ± 0.10	0.50 ± 0.16	-0.34	-0.41	-0.26	-9.47	0.001	2.55

SD, standard deviation; MD, mean difference; CI, confidence interval; p-value, probability value.

### Comparison of contraction ratio of the erector spinae and multifidus between affected and non affected sides of study group

There was a significant reduction in contraction ratio of the ES and LM of the affected side compared with that of the non-affected side (p < 0.05) (Table 4).

**Table 4:** Comparison of contraction ratio of the erector spinae, and multifidus muscles between affected and non affected sides of study group:

Contraction Ratio	Affected Side	Non affected Side		95% CI				
	Mean ± SD	Mean ± SD	MD	Lower Limit	**Upper** **limit**	t- value	p value	Effect Size
Erector spinae	0.11 ± 0.08	0.39 ± 0.09	-0.28	-0.32	-0.24	-13.83	0.001	3.29
Multifidus	0.12 ± 0.07	0.16 ± 0.10	-0.04	-0.08	-0.01	-2.33	0.02	0.46

SD, standard deviation; MD, mean difference; CI, confidence interval; p-value, probability value.

## Discussion

The current study investigated the changes in the contraction ratio of the LM and ES muscles among patients with unilateral SIJ pain and healthy subjects.

The findings showed a trend of reduction in the contraction ratio of the LM and ES muscles on the side of SIJ pain when compared with the contralateral side, as well as against matched healthy individuals. However, in healthy individuals, a significant increase was observed in the contraction ratio of both the LM muscle and the ipsilateral ES when compared with the patients who had SIJ pain.

Several previous investigations have found that individuals with lumbopelvic pain delayed the muscle activity of the ES and LM^25–27^, which supports the lower contraction ratio of the LM and ES.

Furthermore, Heidari (2015)^28^ discovered LM muscle weakness in the lower area of the spine and asymmetry in patients with unilateral discomfort, as shown by smaller increments in thickness on RUSI images during contraction compared to contralateral normal side muscle or asymptomatic control participants. Our findings contradicted Wattananon 2019^29^, who stated that individuals with nonspecific LBP displayed enhanced ipsilateral ES muscle activation to lessen this shear load on the lumbar spine and replace the hypoactivity of LM.

The current observation might be explained by the fact that lumbopelvic stabilizers operate together to form a hard cylinder in the abdominal cavity, absorbing the mechanical stress on the sacroiliac joint and aiding in proper load transfer to the pelvis and lower extremities. The lowered thickness and contraction ratio of the muscles may influence the biomechanical properties of the joint by modifying mechanical stress and load^30^. This method frequently fails in chronic lumbopelvic patients who have limited motor control, high levels of stress, increased or perceived risk of discomfort, and/or a lack of posture awareness and demand^31^.

The LM and ES muscles are the most important muscle groups for stabilizing, loading, and extending the spine and pelvis. The sacral links of the ES and LM complex cause nutation in the SIJ, tensing the stabilizing ligaments. These muscles serve a dual purpose since their iliac connections pull the posterior surfaces of the iliac bones together, limiting nutation. This means that during nutation, the ES and LM cause the cranial side of the SIJ to compress while the caudal side widens or gaps^32^. Strain on the SIJ can result from asymmetric forces operating on the joint, such as a higher unilateral pull caused by the suppression of one side’s ES and/or LM muscles compared to the other^33^.

In unilateral SIJ pain, localized, bilateral multifidus atrophy may be seen as a weakened size of the multifidus, which will probably decrease its capacity to control intersegmental motion, increasing vulnerability to further injury, while there are no significant differences in ES muscle. This can be interpreted by the disuse pattern of the deep stabilizing multifidus, which is compensated for by an overactive ES muscle^34^. While under normal settings, dominant LM has been shown to be greater in some athletes due to the nature of their training^35^.

Healthy individuals have moderate degrees of co-activation of the paraspinal and abdominal muscles and provide adequate spine stability. Altered motor control of deeper stabilizing muscles plays a role in lumbopelvic pain and helps because they provide truncal stabilization through increased force closure of spinal elements and SIJs; additionally, they have anticipatory stabilizing ability, being activated prior to gross movements with relatively greater predictability and lower loads^36^.

The multifidus muscles, together with the transversus abdominis and pelvic floor muscles, constitute the anatomical girdle. These deep compartment muscles are critical for maintaining spinal stability^37^.

### Limitations and strength

One of the study’s primary limitations was that the examination of other neighboring muscles, such as the abdominal and gluteal muscles, might provide more information about the contraction ratio in individuals with SIJ pain.

This is the only study that investigates LM and ES contraction in unilateral SIJ pain and compares them to the unaffected side and healthy participants. Ultrasound imaging has been established as reliable and valid for measuring modifications to muscle thickness, but it remains an indirect approach for assessing muscle activity when compared to EMG. Also, this study did not demonstrate the contraction ratio of LM and ES between side among healthy for confirm that there were symmetry contraction ratio.

## Conclusion

In patients with unilateral sacroiliac joint pain, there is a decrease in the contraction ratio of the erector spinae and multifidus muscles of the affected side when compared to that of the non-affected side and an increase in the contraction ratio of the erector spinae and multifidus of the ipsilateral (dominant) side and the contraction ratio of the contralateral multifidus muscle of a healthy individual compared to the study group. However, there was no significant difference in the contraction ratio of the contralateral erector spinae of a healthy individual compared with that of the non-affected side of the study group.

## Data Availability

Data will be held with the research author and maybe available upon request from corresponding author (Doaa A. Abdel Hady).
